# Effect of a mobile phone-based intervention on post-abortion contraception: a randomized controlled trial in Cambodia

**DOI:** 10.2471/BLT.15.160267

**Published:** 2015-10-15

**Authors:** Chris Smith, Thoai D Ngo, Judy Gold, Phil Edwards, Uk Vannak, Ly Sokhey, Kazuyo Machiyama, Emma Slaymaker, Ruby Warnock, Ona McCarthy, Caroline Free

**Affiliations:** a Department of Population Health, London School of Hygiene & Tropical Medicine, Keppel Street, London, WC1E 7HT, England.; b Innovations for Poverty Action, New Haven, United States of America.; c Independent consultant, London, England.; d Marie Stopes International, Phnom Penh, Cambodia.

## Abstract

**Objective:**

To assess the effect of a mobile phone-based intervention (mHealth) on post-abortion contraception use by women in Cambodia.

**Methods:**

The Mobile Technology for Improved Family Planning (MOTIF) study involved women who sought safe abortion services at four Marie Stopes International clinics in Cambodia. We randomly allocated 249 women to a mobile phone-based intervention, which comprised six automated, interactive voice messages with counsellor phone support, as required, whereas 251 women were allocated to a control group receiving standard care. The primary outcome was the self-reported use of an effective contraceptive method, 4 and 12 months after an abortion.

**Findings:**

Data on effective contraceptive use were available for 431 (86%) participants at 4 months and 328 (66%) at 12 months. Significantly more women in the intervention than the control group reported effective contraception use at 4 months (64% versus 46%, respectively; relative risk, RR: 1.39; 95% confidence interval, CI: 1.17–1.66) but not at 12 months (50% versus 43%, respectively; RR: 1.16; 95% CI: 0.92–1.47). However, significantly more women in the intervention group reported using a long-acting contraceptive method at both follow-up times. There was no significant difference between the groups in repeat pregnancies or abortions at 4 or 12 months.

**Conclusion:**

Adding a mobile phone-based intervention to abortion care services in Cambodia had a short-term effect on the overall use of any effective contraception, while the use of long-acting contraceptive methods lasted throughout the study period.

## Résumé

**Objectif:**

Évaluer l'effet d'une intervention par téléphone portable (santé sur mobile) sur l’utilisation de méthodes contraceptives après un avortement par les femmes au Cambodge.

**Méthodes:**

L'étude MOTIF (Mobile Technology for Improved Family Planning – Technologie mobile pour une meilleure planification familiale) a mobilisé des femmes ayant eu recours à des services d'avortement médicalisé dans quatre cliniques Marie Stopes International du Cambodge. Nous avons aléatoirement affecté 249 femmes au groupe bénéficiant d'une intervention par téléphone portable, laquelle comprenait six messages vocaux automatisés et interactifs et, au besoin, l'assistance téléphonique d'un conseiller, et 251 femmes au groupe de contrôle recevant une prise en charge standard. Le critère d'évaluation principal était l'utilisation autodéclarée d'une méthode contraceptive efficace, 4 et 12 mois après un avortement.

**Résultats:**

Les données relatives à l'utilisation de contraceptifs efficaces étaient disponibles pour 431 (86%) participantes à 4 mois et pour 328 (66%) à 12 mois. Les femmes appartenant au groupe ayant bénéficié de l’intervention sont beaucoup plus nombreuses que les femmes du groupe de contrôle à avoir mentionné l'utilisation de contraceptifs efficaces à 4 mois (64% pour les premières contre 46% pour les secondes; risque relatif, RR: 1,39; intervalle de confiance, IC, à 95%: 1,17-1,66), ce qui n’est pas le cas à 12 mois (50% contre 43%; RR: 1,16; IC à 95%: 0,92-1,47). Cependant, un nombre beaucoup plus élevé de femmes ayant bénéficié de l’intervention a indiqué avoir utilisé une méthode contraceptive à long terme sur ces deux périodes de suivi. Aucune différence notable n'a été constatée entre les deux groupes concernant des grossesses ou des avortements répétés à 4 ou 12 mois.

**Conclusion:**

L'adjonction d'une intervention par téléphone portable aux services de soins après avortement au Cambodge a produit un effet à court terme sur l'utilisation générale de contraceptifs efficaces, tandis que l'utilisation de méthodes contraceptives à long terme s'est poursuivie tout au long de la période d'étude.

## Resumen

**Objetivo:**

Evaluar el efecto de una intervención basada en la telefonía móvil (mHealth) en el uso de métodos anticonceptivos postaborto entre las mujeres de Camboya.

**Métodos:**

El estudio Mobile Technology for Improved Family Planning (MOTIF) involucró a mujeres que recurrieron a servicios de aborto seguros en cuatro clínicas Marie Stopes International de Camboya. Se asignaron de forma aleatoria 249 mujeres a una intervención basada en la telefonía móvil que consistía en seis mensajes de voz interactivos y automatizados y el apoyo de un asesor a través del teléfono, cuando fuera necesario, y 251 mujeres a un grupo de control que recibía atención estándar. El resultado principal fue el uso autodeclarado de un método anticonceptivo efectivo 4 y 12 meses después de sufrir un aborto.

**Resultados:**

Los datos sobre el uso efectivo de los métodos anticonceptivos estuvieron disponibles al cabo de 4 meses en el caso de 431 (86%) de las participantes y al cabo de 12 meses en el caso de 328 (66%). Significativamente más mujeres del grupo de la intervención que del grupo de control informaron de un uso efectivo de los métodos anticonceptivos al cabo de 4 meses (64% frente a 46% respectivamente; riesgo relativo, RR: 1,39; intervalo de confianza (IC) del 95%: 1,17–1,66) pero no al cabo de 12 meses (50% frente a 43% respectivamente; RR: 1,16 (IC del 95%: 0,92–1,47). Sin embargo, más mujeres del grupo de la intervención informaron sobre el uso de métodos anticonceptivos de larga duración en los dos momentos del seguimiento. No hubo una diferencia importante entre los grupos en cuanto a embarazos futuros o abortos al cabo de 4 o 12 meses.

**Conclusión:**

Añadir una intervención basada en la telefonía móvil a los servicios de atención al aborto en Camboya tuvo un efecto a corto plazo en el uso general de cualquier método anticonceptivo efectivo, mientras que el uso de métodos anticonceptivos a largo plazo perduró a lo largo de todo el periodo de estudio.

## ملخص

**الغرض:**

تقييم تأثير التدخل القائم على الهاتف الجوّال (mHealth) على استخدام النساء لموانع الحمل بعد الإجهاض في كمبوديا.

**الطريقة:**

تضمنت دراسة استخدام تكنولوجيا الهاتف الجوّال لتحسين تنظيم الأسرة (‏MOTIF‏) النساء اللاتي سعين إلى الحصول على خدمات الإجهاض الآمن في أربع من عيادات "ماري ستوبس الدولية" في كمبوديا. ولقد قمنا بتخصيص 249 امرأة على نحو عشوائي لإجراء التدخل القائم على الهاتف الجوّال، الذي يتألف من ست رسائل صوتية آلية وتفاعلية مع خدمة الدعم الهاتفي للمرشد العلاجي، عند الضرورة، في حين تم تخصيص 251 امرأة إلى مجموعة شاهدة تتلقى مستوى معياريًا من الرعاية. وكانت المحصلة الأساسية هي الإبلاغ الذاتي عن استخدام وسيلة فعالة لمنع الحمل بعد مرور 4 شهور و12 شهرًا من الإجهاض.

**النتائج:**

توفرت البيانات عن استخدام موانع الحمل الفعالة لعدد 431 من المشاركات (بنسبة 86%) بعد مرور 4 شهور و328 من المشاركات (بنسبة 66%) بعد مرور 12 شهرًا. وبشكل ملحوظ، قامت الكثير من النساء المشاركات في مجموعات التدخل عن النساء المشاركات في المجموعة الشاهدة بالإبلاغ عن استخدام موانع حمل فعالة بعد مرور 4 شهور (بنسبة 64% مقابل 46%، على التوالي؛ الاختطار النسبي: 1.39؛ بنسبة أرجحية مقدارها 95%: 1.17–1.66) ولكن ليس بعد مرور 12 شهرًا (بنسبة 50% مقابل 43%، على التوالي؛ الاختطار النسبي: 1.16؛ بنسبة أرجحية مقدارها 95%: 0.92–1.47). ومع ذلك، أبلغت الكثير من النساء المشاركات في مجموعة التدخل عن استخدام وسيلة لمنع الحمل طويلة المفعول في كلٍ من المرتين اللتين تمت فيهما المتابعة. لم يكن هناك فرق كبير بين المجموعتين في حالات الحمل أو الإجهاض المتكررة بعد مرور 4 شهور أو 12 شهرًا.

**الاستنتاج:**

أدت إضافة التدخل القائم على الهاتف الجوّال إلى خدمات الرعاية بعد الإجهاض في كمبوديا إلى تحقيق تأثير قصير الأجل على الاستخدام العام لأي موانع حمل فعالة، في حين استمر استخدام وسائل منع الحمل طويلة المفعول طوال فترة الدراسة.

## 摘要

**目的:**

旨在评估基于手机的干预（移动医疗）对柬埔寨女性流产后避孕的影响。

**方法:**

面向在柬埔寨的四家玛丽斯特普国际组织 (Marie Stopes International) 下属诊所中寻求安全流产服务的女性，展开改善计划生育的移动技术 (MOTIF) 研究。我们随机分配 249 位女性进行基于手机的干预，其包括带有顾问电话支持的 6 个自动化、交互式语音信息，而另外 251 位女性被分配至对照组，接受标准治疗。主要结果为，流产后 4 和 12 个月时自报有效避孕方法的使用情况。

**结果:**

4 个月时，431 位参与者 (86%) 获得有效避孕的使用数据，12 个月时为 328 位 (66%)。4 个月时，报告有效避孕的女性中，参与干预的女性明显超出对照组（64% 比 46%；相对风险率 RR： 1.39；95% 置信区间 CI：1.17-1.66），但 12 个月时却有所不同（分别为 50% 比 43%，相对风险率 RR：1.16；95% 置信区间 CI：0.92–1.47). 然而，在后续两个时间点，干预组中报告使用长效避孕方法的女性明显更多。4 个月或 12 个月时，两组中再次怀孕或流产的人数没有显著差异。

**结论:**

为柬埔寨为流产护理服务添加基于手机的干预，可在短期内对任何有效避孕的总体使用情况产生一定的效果，而整个研究期间则继续使用长效避孕方法。

## Резюме

**Цель:**

Оценить влияние мероприятий здравоохранения, проводимых с помощью мобильной связи (mHealth), на применение способов постабортной контрацепции женщинами, проживающими в Камбодже.

**Методы:**

В исследовании «Применение мобильных технологий для усовершенствованного планирования семьи» (MOTIF)  приняли участие женщины, желающие пройти процедуру безопасного аборта в четырех клиниках Marie Stopes International, Камбоджа. 249 женщин были случайным образом распределены в группу, в которой медицинское обслуживание проводилось посредством мобильной связи и включало возможность отправки шести автоматизированных интерактивных голосовых сообщений с поддержкой телефонной связи с консультантом при необходимости, а 251 женщина была распределена в контрольную группу, в которой участники получали медицинское обслуживание стандартным способом. Первичным результатом являлось применение эффективного способа контрацепции через 4 и 12 месяцев после аборта, о чем участницы сообщали самостоятельно.

**Результаты:**

Данные о применении эффективного способа контрацепции были получены для 431 (86%) участницы через 4 месяца и для 328 (66%) участниц через 12 месяцев. Число женщин, сообщивших о применении эффективного способа контрацепции через 4 месяца, было больше в группе, в которой проводилось обслуживание посредством мобильной связи, чем в контрольной группе (64 против 46% соответственно, относительный риск, ОР: 1,39; 95% доверительный интервал, ДИ: 1,17–1,66), но через 12 месяцев был получен другой результат (50 против 43% соответственно, ОР: 1,16; 95% ДИ: 0,92–1,47). Тем не менее в каждый из двух контрольных моментов времени послеоперационного наблюдения число женщин в группе проведения исследуемого мероприятия, сообщивших о применении способа контрацепции длительного действия, преобладало. Что касается числа повторных беременностей или абортов через 4 или 12 месяцев, значимой разницы между двумя группами выявлено не было.

**Вывод:**

Добавление медицинского обслуживания посредством мобильной связи в рамки медицинской помощи при аборте в Камбодже оказало краткосрочное влияние на общее применение какого-либо эффективного способа контрацепции, в то время как способы контрацепции длительного действия применялись в течение всего исследования.

## Introduction

Unmet need for contraception can result in unintended pregnancy and avoidable maternal and infant deaths.^1^ It has been estimated that, if the need for modern contraception methods were met, 52 million unintended pregnancies, 24 million abortions (over half of which would be unsafe) and 70 000 maternal deaths would be prevented among women in low-income countries each year. Nevertheless, 225 million women in these countries had an unmet need for contraception in 2014.^2^

Women who seek an abortion are likely to have an unmet need for contraception and the time after an abortion provides a key opportunity to offer family planning services.^3^ Typically, women are counselled on family planning before discharge from clinical care after seeking abortion services.^4^ However, quality of service provision varies and evidence on the ability of enhanced counselling interventions to improve post-abortion family planning is inconclusive.^5^^,^^6^

In Cambodia, despite the total fertility rate declining from 3.4 births per woman in 2005 to 3.0 births per woman in 2010, there remains an unmet need for contraception: in 2010, 81% of women of reproductive age reported wanting to delay their next child or to have no more children but only 35% reported currently using a modern contraceptive method.^7^ The abortion rate in the country was estimated to be 50 per 1000 women, compared to a global average of 28 per 1000,^8^ and 26% of women who sought abortion services had had more than one abortion.^7^

Interventions delivered by mobile phone could help increase the uptake and continuation of post-abortion family planning in countries like Cambodia where over 90% of the 2066 women surveyed report owning a mobile phone.^9^ Health interventions delivered by mobile phone can utilize different approaches (e.g. text messages, voice messages or smartphone applications) depending on the literacy of the population and the devices available.^10^ Compared with face-to-face interventions, mobile phone-based interventions have the advantage that they can provide interactive, personalized support inexpensively wherever the person is located and whenever needed. Our research suggested that women in Cambodia often found it difficult to make decisions about contraception at the time of seeking abortion services; they needed more time, to wait for their health to improve or to speak with family or friends.^11^ Hence, in this setting, where 80% of the population live in a rural area and geographical distances can restrict access to services, mobile phone-based interventions may provide an effective method for maintaining communication with clients after they leave the clinic.^7^^,^^12^ Interventions delivered by mobile phone have been shown to be effective in other health areas, such as smoking cessation and adherence to treatment for human immunodeficiency virus infection.^13^^,^^14^ However, the evidence from three small trials in which a mobile phone-based intervention was used to increase contraceptive use has been inconclusive.^15^^–^^17^ The objective of our study was to evaluate the effectiveness of a mobile phone-based intervention designed to support post-abortion contraception in Cambodia. The specific aims were to increase the uptake of effective contraceptive methods and to reduce contraceptive discontinuation.

## Methods

Our study – the Mobile Technology for Improved Family Planning (MOTIF) study – was a single-blind, randomized trial of a personalized, mobile phone-based intervention designed to support post-abortion family planning. The protocol was published in 2013.^18^^,^^19^ The trial was undertaken at four Marie Stopes International clinics in Cambodia that provided safe abortion services: two served peri-urban populations around Phnom Penh city (i.e. Chbar Ambov and Takmao) and two served provincial towns with a predominantly rural population (i.e. Battambang and Siem Reap). All women older than 17 years who sought an induced abortion were eligible for inclusion if they had a mobile phone primarily for their own use, reported not wanting to become pregnant and were willing to receive automated voice messages about contraception. Research assistants interviewed women after they had received post-abortion family planning counselling at the clinic to assess their eligibility for the study and to collect baseline data. Participants provided consent by written signature or thumbprint. Ethical approval was obtained from ethics committees at the London School of Hygiene & Tropical Medicine and Marie Stopes International and the Cambodia Human Research ethics committee. The trial was registered through ClinicalTrials.gov with the identifier NCT01823861.

Research assistants provided a written list of participants, each with a unique identification number, to counsellors delivering the intervention. The project statistician at the London School of Hygiene & Tropical Medicine, London, United Kingdom of Great Britain and Northern Ireland, received only the identification number and the urban or rural clinic classification of each participant. The statistician allocated participants to the intervention or control group on a 1:1 basis using Minim (https://www-users.york.ac.uk/~mb55/guide/minim.htm), a computer randomization program that stratified them according to whether their clinic was urban or rural. The identification numbers of participants allocated to the intervention were sent to the counsellors between 1 May and 27 September 2013. Researchers who undertook data collection and analysis were blinded to the treatment allocation.

All participants received existing standard care, which included post-abortion family planning counselling at the clinic in accordance with national guidelines, the offer of a follow-up appointment at the clinic and details of the clinic’s phone number and of a hotline number operated by counsellors at Marie Stopes International Cambodia. Those allocated to the intervention, which lasted 3 months, also received six automated, interactive voice messages and were provided with phone support from a counsellor depending on their responses to the messages (Box 1). Participants who chose to receive oral or injectable contraceptives could opt for additional reminder phone messages appropriate to their method. Participants in the control group did not receive voice messages. The formative research carried out to develop the intervention will be reported elsewhere.

Box 1 **The mobile phone-based intervention**

The conceptual framework for the intervention used in the MObile Technology for Improved Family Planning (MOTIF) study was based on literature reports on the determinants of contraceptive use and on links between contraceptive use and fertility.^18^ The intervention comprised six automated voice messages sent to participants’ mobile phones, at the time of their preference, during the 3 months following an abortion. Participants received the first message within 1 week of using abortion services and every 2 weeks thereafter. The message, recorded in the Khmer language, was as follows:

Hello, this is a voice message from a Marie Stopes counsellor. I hope you are doing fine. Contraceptive methods are an effective and safe way to prevent an unplanned pregnancy. I am waiting to provide free and confidential contraceptive support to you. Press 1 if you would like me to call you back to discuss contraception. Press 2 if you are comfortable with using contraception and you do not need me to call you back this time. Press 3 if you would prefer not to receive any more messages.

Participants who pressed 1 or who did not respond received a phone call from a counsellor. The phone calls were intended to encourage contraceptive use by increasing the client’s capability of using contraception by: (i) providing individualized information on a range of contraceptive methods; (ii) increasing the participant’s opportunity to use contraception, for example, by informing her where she could access specific methods near her residence; and (iii) increasing motivation by reinforcing knowledge of the benefits of contraception. At the participant’s request, the counsellor would also discuss contraception with her husband or partner.

Participants were also able to call the service and ask to speak to a counsellor. Those who chose to receive an oral or injectable contraceptive could opt to receive additional reminder messages appropriate to their method (e.g. on when to start a new packet of pills or when to receive a new injection). The sixth and final voice message was similar but also reminded the participant that this was the last message they would receive.

The intervention was delivered by trained counsellors at Marie Stopes International Cambodia. Voice messages were scheduled and sent using the open-source software program Verboice (InSTEDD, Palo Alto, United States of America). The cost of outgoing communications from the provider to the participant was met by Marie Stopes International Cambodia and the cost of calling into the service (i.e. a local call) was incurred by participants.

The primary outcome was the self-reported use of an effective contraception method, 4 and 12 months after an abortion. Effective methods were defined as those that have been associated with a 12-month pregnancy rate below 10% (a common criterion in developing countries), such as oral contraceptives, 3-monthly contraceptive injections, subdermal implants, intrauterine devices and permanent methods, such as sterilization or vasectomy.^20^^,21^ A participant was regarded as using an effective method if she reported that she: (i) currently had a contraceptive implant or an intrauterine device in place; (ii) had received a contraceptive injection within the previous 3 months; (iii) had undergone sterilization or her husband or partner had had a vasectomy; or (iv) had taken an oral contraceptive within 24 hours of the interview or according to instructions. Secondary outcomes were: (i) use of a long-acting contraceptive method (i.e. an intrauterine device, implant or permanent method); (ii) repeat pregnancy; (iii) repeat abortion; (iv) effective contraceptive use for more than 80% of the 4 or 12 months after the abortion; (v) road traffic accidents associated with the intervention (e.g. caused by driving while using the phone); and (vi) domestic abuse associated with the intervention (e.g. after the woman’s husband or partner had listened to the messages). Research assistants contacted participants by phone and collected information on these outcomes using a standardized questionnaire. The effect of the intervention was examined in prespecified subgroups categorized by age, urban or rural residence, educational level and socioeconomic status – access to a motorized vehicle was used as a proxy measure of socioeconomic status. The 4-month follow-ups were conducted between 13 August 2013 and 31 January 2014 and the 12-month follow-ups, between 24 July and 16 November 2014. An assessment of the validity of the self-reported data collected after 4 months in 50 participants will be reported elsewhere.

### Statistical analysis

The statistical analysis plan was specified before the study was unblinded and was reported in the trial protocol.^18^ We estimated that 35% of the control group would be using an effective contraception method after 4 months and that a sample size of 500 would be required to detect a 13% increase in contraceptive use with a 90% power at the 5% level of significance.^18^ Analyses were undertaken on an intention-to-treat basis using Stata version 13.1 (StataCorp. LP, College Station, United States of America). The effect of the intervention was expressed as a relative risk (RR) or hazard ratio (HR); a 95% confidence interval (CI) was used for primary and secondary outcomes and a 99% CI for subgroup analyses. The contraceptive discontinuation rate was assessed using Kaplan–Meier survival analysis techniques: for 4-month follow-up data, discontinuation was assessed in participants who started using an effective contraception method during the first 4 weeks after an abortion and, for 12-month follow-up data, discontinuation was assessed in those who started the method during the 3 months after an abortion. Discontinuation was defined as stopping the method for 1 week or more before the 4-month follow-up or for 1 month or more before the 12-month follow-up. If the participant switched from one effective method to another effective method this was not considered discontinuation.

## Results

**Fig. 1:**
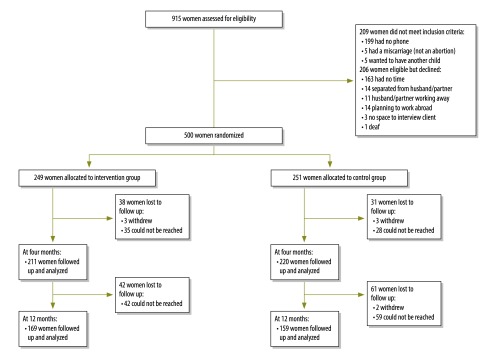
Flowchart of participants in a mobile phone-based intervention for post-abortion contraception, Cambodia, 2013–2014

Note: The intervention comprised six automated, interactive voice messages by mobile phone and phone support from a counsellor, as required, during the 3 months following an abortion (Box 1).

We excluded 199 potential participants because they did not own a mobile phone. Of the 500 participants, 249 were assigned to the intervention group and 251 to the control group (Fig. 1). The participants’ baseline characteristics are shown in Table 1. Data on the primary outcome were available for 431 (86%) participants at 4 months and for 328 (66%) at 12 months. Over 75% (133/172) of losses to follow-up by 12 months were due to the participant’s phone being either switched off or not in use, as indicated by an automated message. Less frequently the phone number had been reassigned to another user or the participant had reportedly moved abroad for work. The proportion of women in the intervention group who reported effective contraception use was significantly higher than in the control group at 4 months (64% versus 46%, respectively; RR: 1.39; 95% CI: 1.17–1.66; Table 2) but not at 12 months (50% versus 43%, respectively; RR: 1.16; 95% CI: 0.92–1.47).

**Table 1:** Baseline characteristics of participants in a mobile phone-based intervention for post-abortion contraception, Cambodia, 2013–2014

Characteristic	Intervention group (*n* = 249)	Control group (*n* = 251)
	No. (%)	No. (%)
**Age, years**		
< 25	88 (35)	69 (27)
≥ 25	161 (65)	182 (73)
**Residence**		
Rural	164 (66)	157 (63)
Urban	85 (34)	94 (37)
**Educational level**		
None or primary school	93 (37)	103 (41)
Secondary school or higher	156 (63)	148 (59)
**Socioeconomic status**		
Access to a motorized vehicle	221 (89)	214 (85)
No access to a motorized vehicle	28 (11)	37 (15)
**Marital status**		
Married or cohabiting	231 (93)	233 (93)
Never married or cohabited	15 (6)	14 (6)
Divorced or separated	3 (1)	4 (2)
**Literacy**		
Able to recognize numbers	246 (99)	250 (100)
Not able to recognize numbers	3 (1)	1 (> 1)
**Number of living children**		
0	79 (32)	68 (27)
1 or 2	122 (49)	131 (52)
≥ 3	48 (19)	52 (21)
**Previous abortions**		
0	144 (58)	155 (62)
1	69 (28)	65 (26)
≥ 2	36 (15)	31 (12)
**Type of abortion before study entry**		
Medical	102 (41)	105 (42)
Surgical	147 (59)	146 (58)
**Woman planned to use contraception at time of randomization**		
Yes	91 (37)	96 (38)
No	18 (7)	24 (10)
Undecided	140 (56)	131 (52)
**Woman’s mobile phone access**		
Shares phone	123 (49)	118 (47)
Never shares phone	126 (51)	133 (53)

Note: The intervention comprised six automated, interactive voice messages by mobile phone and phone support from a counsellor, as required (Box 1). Inconsistencies arise in some values due to rounding.

**Table 2:** Effect of mobile phone-based intervention^a^ on post-abortion contraception, Cambodia, 2013–2014

Outcome	Four-month follow-up				Twelve-month follow-up		
	Intervention group	Control group	RR (95% CI)		Intervention group	Control group	RR (95% CI)
	No./total no. of respondents (%)	No./total no. of respondents (%)			No./total no. of respondents (%)	No./total no. of respondents (%)	
**Primary outcome**							
Self-reported use of an effective contraceptive method	135/211 (64)	101/220 (46)	1.39 (1.17–1.66)		84/169 (50)	68/159 (43)	1.16 (0.92–1.47)
**Secondary outcome**							
Use of a long-acting contraceptive method	61/211 (29)	19/220 (9)	3.35 (2.07–5.40)		42/169 (25)	19/159 (12)	2.08 (1.27–3.42)
Effective contraceptive use for > 80% of the follow-up period	108/200 (54)	81/203 (40)	1.35 (1.10–1.67)		86/169 (51)	61/159 (38)	1.33 (1.04–1.70)
Contraceptive discontinuation	9/123 (7)	16/101 (16)	0.45^a^ (0.20–1.01)		28/107 (26)	25/83 (30)	0.82^b^ (0.48–1.40)
Repeat pregnancy	6/210 (3)	5/220 (2)	1.25 (0.39–4.06)		22/169 (13)	28/159 (18)	0.74 (0.44–1.24)
Repeat abortion	2/210 (1)	1/220 (0.5)	2.10 (0.19–22.9)		8/169 (5)	11/159 (7)	0.68 (0.28–1.66)
Involvement in a road traffic accident	0/210 (0)	0/220 (0)	NA		ND	ND	NA
Experience of domestic abuse	0/210 (0)	0/220 (0)	NA		ND	ND	NA
Lost to follow-up^b^	38/249 (15)	31/251 (12)	1.24 (0.80–1.92)		80/249 (32)	92/251 (37)	0.88 (0.69–1.12)
Withdrawal from study	3/249 (1)	3/251 (1)	1.01 (0.21–4.95)		3/249 (1)	5/251 (2)	0.60 (0.15–2.50)

CI: confidence interval; NA: not applicable; ND: not determined; RR: relative risk.

^a^ The value for contraceptive discontinuation is the hazard ratio, not the relative risk.

^b^ The number lost to follow-up includes participants who withdrew from the study.

Note: The intervention comprised six automated, interactive voice messages by mobile phone and phone support from a counsellor, as required, during 3 months following an abortion (Box 1).

**Fig. 2:**
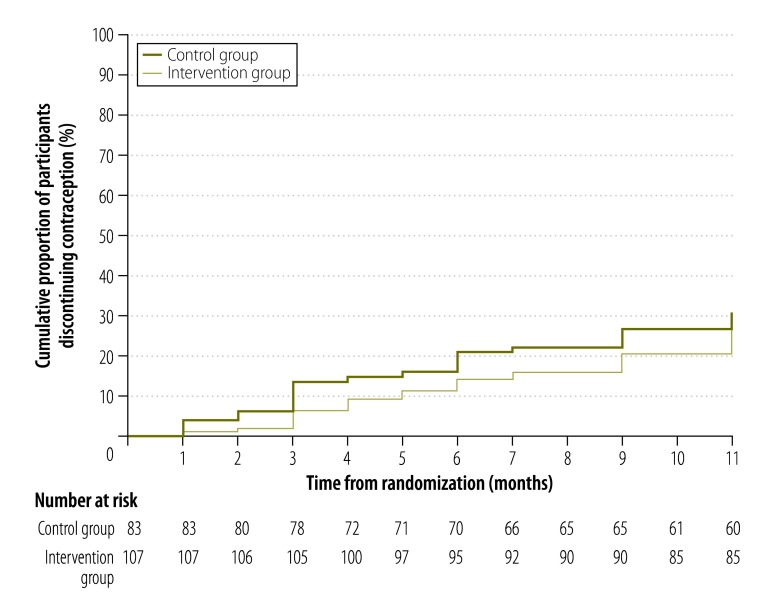
Kaplan–Meier survival curves for contraceptive discontinuation, in a mobile phone-based intervention for post-abortion contraception, Cambodia, 2013–2014

Note: The intervention comprised six automated, interactive voice messages by mobile phone and phone support from a counsellor, as required, during 3 months following an abortion (Box 1). Discontinuation, defined as stopping for ≥ 1 month, was assessed at 12 months in the 190 participants who started an effective method of contraception in the 3 months after an abortion. At the 12-month follow up, data were collected on self-reported contraception use during each month following the abortion. The number at risk is the number of contraception users at each time point.

**Fig. 3:**
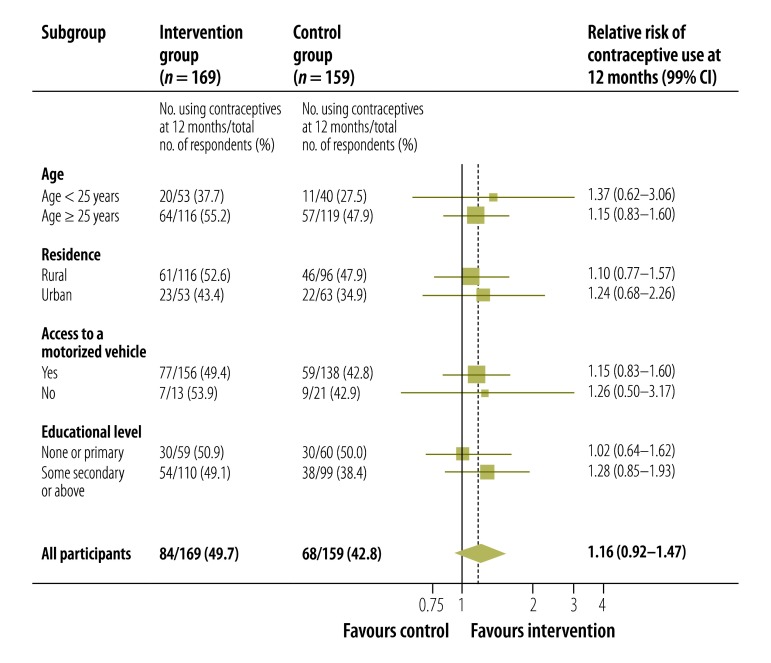
Contraceptive use in different subgroups after a mobile phone-based intervention for post-abortion contraception, Cambodia, 2013–2014

Significantly more women in the intervention than the control group reported using a long-acting contraceptive method at 4 months (29% versus 9%, respectively; RR: 3.35; 95% CI: 2.07–5.40; Table 2) and at 12 months (25% versus 12%, respectively; RR: 2.08; 95% CI: 1.27–3.42). In addition, significantly more women in the intervention than the control group reported effective contraceptive use for more than 80% of the 4 months after the abortion (54% versus 40%, respectively; RR: 1.35; 95% CI: 1.10–1.67) and for more than 80% of the 12 months after (51% versus 38%, respectively; RR: 1.33; 95% CI: 1.04–1.70). There was some evidence that fewer women in the intervention than the control group had discontinued contraceptive use by the 4-month follow-up (7% versus 16%, respectively; HR: 0.45; 95% CI: 0.20–1.01; Table 2) but not by the 12-month follow-up (26% versus 30%, respectively; HR: 0.82; 95% CI: 0.48–1.40; Fig. 2, available at: http://www.who.int/bulletin/volumes/93/12/15-160267). There was no significant difference between the groups in the proportion of women who had a repeat pregnancy or an abortion by 4 or 12 months and there were no reports that the intervention had been associated with a road traffic accident or domestic abuse at 4 months (Table 2). The subgroup analysis found no evidence that either age, urban or rural residence, educational level or socioeconomic status influenced the effect of the intervention on contraception use at 12 months (Fig. 3).

CI: confidence interval.

Note: The intervention comprised six automated interactive voice messages by mobile phone and phone support from a counsellor, as required, during 3 months following an abortion (Box 1). Contraceptive use was defined as the self-reported use of an effective contraception method 12 months after an abortion.

## Discussion

Our mobile phone-based intervention was associated with an increase in the self-reported use of an effective contraceptive method 4 months after an abortion but not 12 months after. However, more participants in the intervention than the control group reported using a long-acting contraceptive method at 4 and 12 months. The intervention had no significant effect on the repeat pregnancy or abortion rate and there were no reports of adverse effects.

This study has several strengths. First, all analyses were carried out on an intention-to-treat basis. Few trials of post-abortion family planning have a longer observation period than our study.^5^ The follow-up rate at 4 months was high and there was no evidence of any difference in losses to follow-up between the treatment groups. However, the follow-up rate at 12 months was only 66%, which decreased the statistical power of our assessment of the long-term effects of the intervention. This low rate was probably due to participants migrating for work and/or changing phone numbers, which is recognized as a challenge for mobile phone-based interventions in Cambodia.^22^

One limitation of the study was that, since the intervention involved behavioural change, it was not possible to blind participants to their treatment allocation and they may have passed on information to the research assistants at follow-up. In addition, the use of self-report measures of contraceptive use has the potential for detection bias. Although they are standard in contraceptive research, self-report measures have been shown to overestimate contraceptive use and underestimate abortion rates.^23^ However, it seems unlikely that participants would over-report using one particular long-acting method rather than another (e.g. intrauterine devices versus implants). It was not feasible to measure objective contraception use in this setting and electronic medication monitors and hormonal assays have limited reliability and validity.^24^^,^^25^ Oral and injectable contraceptives can be obtained from pharmacists without prescriptions in Cambodia, so clinic records may not accurately reflect contraceptive use. The most commonly reported reason for ineligibility was not having a mobile phone. Although we did not record the characteristics of the 199 potential participants in our study who did not have a phone, it is a concern that mobile phone-based interventions may not reach the people most in need. We did not give participants mobile phones because of possible implications for the sustainability of the intervention and because there could have been negative consequences for a participant if she was asked where she obtained a new phone. Study participants were similar to clients seeking abortion services at the four Marie Stopes International Cambodian study clinics during 2013. Most of these women are married and multiparous, had attended secondary school, are aged over 25 years and have previously paid for reproductive health services at a clinic run by a nongovernmental organization. However, sex workers – known to have a high unmet need for contraception and a high abortion rate^26^ – and young women, were not well represented in our study population. The effect of mobile phone-based interventions on post-abortion family planning among these groups requires further evaluation.

There are few trials of mobile phone-based interventions to increase contraception use. Two small trials found no effect,^16^^,^^17^ whereas one trial found improved self-reported adherence to oral contraceptive use.^15^

Service providers often define post-abortion family planning as the initiation of contraceptive use within 2 weeks of an abortion but we did not identify any trials reporting follow-up at this time point. We decided to assess contraception use at 4 months, after the intervention had been completed, because we recognized that side-effects and discontinuation are common in the first few months.^27^ The 12-month follow-up was intended to assess the long-term effects of the intervention. Although at 12 months there was no evidence of increased contraceptive use overall or of less frequent discontinuation, our intervention was associated with an increase in the use of long-acting contraceptive methods in a context where a wide range of post-abortion family planning methods is available but the immediate uptake of contraception is low (data available from corresponding author). The increase occurred because participants returned to the clinic for a contraceptive implant or an intrauterine device and is consistent with our findings that some clients preferred to make decisions about post-abortion family planning after discharge from clinical care.^11^ As our intervention was complex, it was not clear which component influenced the uptake of long-acting methods. It is plausible, though, that a relatively intensive intervention delivered over a short period of time could influence the decision to adopt a long-acting method (i.e. a single behavioural change) but be less effective in influencing continued adherence to an oral contraceptive, which requires sustained repetitive behaviour. In fact, the literature suggests that interventions encouraging medication adherence are more effective for short-term rather than long-term treatments. Furthermore, long-acting contraceptive methods are associated with lower discontinuation rates than short-acting hormonal methods.^27^^–^^29^ We plan to publish a separate report on the results of qualitative interviews with participants about their experience of the intervention.

Few studies have examined contraceptive use for an extended period after an abortion in a low-income setting. One matched, controlled study in Zimbabwe assessed the effect of counselling and free contraception before hospital discharge. At 12 months, effective contraceptive use was higher in the intervention than the control group (84% (227/271) versus 64% (165/258), respectively; *P* < 0.001) and repeat unintended pregnancy was lower (15% (42/276) versus 34% (96/281), respectively; *P* <  0.001) but repeat abortions were not significantly lower (3% versus 5%; *P* = 0.23).^30^ At 12 months in our study, 13% (22/169) of participants in the intervention group reported a repeat pregnancy compared with 18% (28/159) in the control group; the corresponding figures for a repeat abortion were 5% (8/169) and 7% (11/159), respectively. However, the study was not powered to detect differences in these outcomes. Nevertheless, the increased use of long-acting methods and the increased duration of all effective contraceptive use would be expected to result in a decrease in unintended pregnancies and repeat abortions over time. A larger study may be able to detect differences in these outcomes.

## Conclusion

Our results indicate that the addition of a mobile phone-based intervention to existing abortion care services could increase the use of long-acting contraceptives. The overall use of effective contraceptive methods was increased 4 months after an abortion but not at 12 months. In practice, the duration, language and mode of communication (i.e. text or voice) could be adapted to different settings, though voice messages will be most useful in populations with limited literacy. We estimated the main cost of delivering the intervention (i.e. for voice messages, phone calls and the counsellors’ time) to be 6 United States dollars per client. A cost–effectiveness analysis will be reported elsewhere. Although our intervention was delivered in addition to post-abortion family planning support at a clinic, future research could assess the effect of a similar intervention in settings with more limited support: for example, where medical abortions are provided by the private sector.
